# Responsiveness and minimal clinically important difference of the Minnesota living with heart failure questionnaire

**DOI:** 10.1186/s12955-019-1104-2

**Published:** 2019-02-14

**Authors:** M. Gonzalez-Saenz de Tejada, A. Bilbao, L. Ansola, R. Quirós, L. García-Perez, G. Navarro, A. Escobar

**Affiliations:** 1 Research Unit, Basurto University Hospital, Jado 4th floor, Avda Montevideo 18, 48013 Bilbao, Vizcaya Spain; 2 Red de Investigación en Servicios de Salud en Enfermedades Crónicas (REDISSEC), Madrid, Spain; 3 Hospital Costa del Sol, Carretera Nacional 340, km 186, Marbella, Málaga, Spain; 4 Planning and Evaluation Service, Canary Islands Health Service, Camino Candelaria, 44 C.S. San Isidro-El Chorrillo, 38109 El Rosario, Tenerife, Spain; 5 Epidemiology Unit, Hospital Universitari, Parc Taulí 1, 08208 Sabadell, Barcelona, Spain

**Keywords:** Minnesota living with heart failure questionnaire, Heart failure, Health-related quality of life, Responsiveness, Minimal clinically important difference, Psychometric properties

## Abstract

**Background:**

The Minnesota Living with Heart Failure Questionnaire (MLHFQ) is one of the most widely used health-related quality of life questionnaires for patients with heart failure (HF). The objective of the present study was to explore the responsiveness of the MLHFQ by estimating the minimal detectable change (MDC) and the minimal clinically important difference (MCID) in Spain.

**Methods:**

Patients hospitalized for HF in the participating hospitals completed the MLHFQ at baseline and 6 months, plus anchor questions at 6 months. To study responsiveness, patients were classified as having “improved”, remained “the same” or “worsened”, using anchor questions. We used the standardized effect size (SES), and standardized response mean (SRM) to measure the magnitude of the changes scores and calculate the MDC and MCID.

**Results:**

Overall, 1211 patients completed the baseline and follow-up questionnaires 6 months after discharge. The mean changes in all MLHFQ domains followed a trend (*P* < 0.0001) with larger gains in quality of life among patients classified as “improved”, smaller gains among those classified as “the same”, and losses among those classified as “worsened”. The SES and SRM responsiveness parameters in the “improved” group were ≥ 0.80 on nearly all scales. Among patients classified as “worsened”, effect sizes were < 0.40, while among patients classified as “the same”, the values ranged from 0.24 to 0.52. The MDC ranged from 7.27 to 16.96. The MCID based on patients whose response to the anchor question was “somewhat better”, ranged from 3.59 to 19.14 points.

**Conclusions:**

All of these results suggest that all domains of the MLHFQ have a good sensitivity to change in the population studied.

## Background

The Heart Failure Association of the European Society of Cardiology defines heart failure (HF) as “a clinical syndrome characterized by typical symptoms (e.g. breathlessness, ankle swelling and fatigue) that may accompanied by signs (e.g. elevated jugular venous pressure, pulmonary crackles and peripheral edema) caused by a structural and/or functional cardiac abnormality, resulting in a reduced cardiac output and/or elevated intracardiac pressures at rest or during stress” [1]. HF is a highly prevalent condition, associated with significant morbidity and a poor prognosis [2–4]. A 2013 update from the American Heart Association estimated that there are 5.1 million people with HF in USA and 23 million worldwide [5]. The incidence of HF also increases significantly with age, and hence, because of the aging population, the prevalence of HF can be expected to increase substantially in the future [6]. In addition, the majority of patients with HF experience a considerable reduction in health-related quality of life (HRQoL) [7].

The HRQoL of patients with HF is an important outcome as it reflects the impact of HF on their daily lives [8]. HF patients experience high levels of physical, functional and emotional distress [9]. Indeed, there is evidence that adults with HF have poorer HRQoL than those without HF [10–13].

In recent decades, various specific HRQoL questionnaires for patients with HF have become regarded as important assessment tools [14–17]. Among these, one of the most widely known and used is the Minnesota Living with Heart Failure Questionnaire (MLHFQ) [16, 18]. It is a self-administered disease-specific questionnaire for patients with HF [19], comprising 21 items. It provides a total score as well as scores for two domains, physical and emotional. The questionnaire has been translated into and validated in Spanish [14].

There are several definitions of measurement responsiveness [20, 21]. For this study, we defined responsiveness as the ability of an instrument to detect real changes in the concept being measured [22]. In the Spanish version of the MLHFQ, responsiveness has not been studied in depth and it is very important to determine whether an HRQoL questionnaire is able detect changes over time in the patient that occur naturally or due to clinical intervention. In addition, HRQoL questionnaires should be easily interpretable by clinicians, and one of the most common ways to facilitate interpretation is ascertaining the minimal detectable change (MDC) and the minimal clinically important difference (MCID) [23]. The latter may vary by patient characteristics or clinical status.

Therefore, the objective of the present study was to examine the responsiveness of the MLHFQ, using distribution and anchor-based approaches [21, 22, 24–26]. Among distribution-based methods, we calculated the MDC at the individual level. We also compared the MDC with the MCID for each domain [23, 27]. To the best of our knowledge, only one previous study has analyzed the responsiveness of the Spanish version of the MLHFQ [14], and our study is the first to explore the responsiveness of this questionnaire by estimating MDC and MCID in Spain.

## Methods

### Sample and materials

Secondary analysis of a prospective, multicenter cohort study, carried out in 13 Spanish hospitals: two in the Canary Islands, one in Catalonia, four in Andalusia and six in the Basque Country. The cohort consisted of hospitalized patients with HF on cardiology or internal medicine departments of the participating hospitals, between January 2009 and May 2013. Within the study period, the first admission was considered the index admission. The Institutional Review Boards of each hospital approved the study. All patients signed a declaration of informed consent.

The inclusion criteria were: having a diagnosis of HF (International Classification of Diseases, 9th Revision*,* Clinical Modification code 428), being more than 18 years of age, completing the questionnaires at baseline and 6 months, and consenting to participate in the study. Patients who developed an HF episode during hospitalization, those referred from other health care centers and those who died during their hospital stay or within 6 months from discharge were excluded from the study.

All eligible patients were sent a letter informing them about the study and asking for their voluntary participation.

Clinical data including smoking history, left ventricular ejection fraction, *New York Heart Association* classification (NYHA) [28] and comorbidities (assessed with Charlson’s Index [29]) were extracted from clinical records. In addition, during admission, patients completed the MLHFQ, plus questions requesting sociodemographic information. At 6 months, we sent the same questionnaires to each participant plus anchor questions. These questions were different for each of the MLHFQ dimensions. For the physical dimension, the anchor question was *“How would you rate your physical problems related to your heart disease compared with how you felt 6 months ago?”* For the emotional dimension the anchor question was *“How would you rate your emotional problems related to your heart disease compared with how you felt 6 months ago?”* The response options for both questions were presented as 5-point ordinal scales (1 = much worse, 2 = somewhat worse, 3 = about the same, 4 = somewhat better, 5 = much better).

The 21 items of MLHFQ [16, 18] are rated on six-point Likert scales, representing different degrees of impact of HF on HRQoL, from 0 (no) to 5 (very much). It provides a total score (range 0–105), from best to worst HRQoL, as well as scores for two subscales, the physical (range 0–40) and emotional (range 0–25) domains, composed of 8 and 5 items respectively. The other eight items (of the total of 21) are only considered for the calculation of the total score. The MLHFQ has been translated and culturally adapted into at least 34 languages, and has demonstrated good psychometric properties in numerous studies [8, 14–16, 30–36].

### Statistical analysis

#### Descriptive statistics

Descriptive data are expressed as frequencies and percentages for qualitative variables and means with standard deviations (SDs) for quantitative variables. We compared sociodemographic and clinical characteristics, and MLHFQ scores at baseline between the responders and non-responders at follow-up using the chi-square test or Student’s t-test.

#### Statistically significant change

To study responsiveness, patients were classified as having “improved”, remained “the same” or “worsened”, according to their anchor question responses. Those who, in response to the anchor questions at 6 months, rated their condition as “much better” or “somewhat better” compared to 6 months earlier were classified as having “improved”, those who rated their condition as “about the same” were classified as having remained “the same”, and patients who rated their condition as “somewhat worse” or “much worse” were classified as having “worsened”. For the total score of MLHFQ, the response was calculated taking into account the answers to the anchor questions of the physical and emotional domains. Specifically, those who rated their condition as “improved” in both anchor questions or “improved” in one and “the same” in other anchor question were classified as “improved”; and those who rated them as “the same” in both anchor questions were classified as “the same”; while those who rated them as “worsened” in either of the anchor questions were classified as “worsened”.

Ceiling and floor effects at baseline and 6 months after discharge were examined to evaluate the discriminatory power of the scales in each subgroup of patients, and we used 15% as the critical value for such effects [37]. Means and SDs were calculated for the MLHFQ scales at baseline and 6 months after discharge in each subgroup of patients, and a paired t-test or the nonparametric Wilcoxon signed-rank test was used to compare scores at the two time points. Changes in MLHFQ scores were calculated by subtracting follow-up scores from baseline scores, a positive result indicating a gain in quality of life. Mean changes were also compared among the three subgroups by analysis of variance with the Scheffe test for multiple comparisons, or the nonparametric Kruskall-Wallis test.

#### Standardized effect size and standardized response mean

To measure the responsiveness of the MLHFQ, we calculated the standardized effect size (SES), defined as the mean change score divided by the SD of the baseline scores, and standardized response mean (SRM), defined as the mean change score divided by the SD of the change scores [22]. Cohen’s benchmarks were used to classify the magnitude of the effect sizes: < 0.20, not significant; 0.20 to 0.49, small; 0.50 to 0.79, moderate; and ≥ 0.80, large [24]. We hypothesized that there would be larger HRQoL changes in patients who rated their condition as better or worse than in patients who considered that they had remained “the same”.

#### MDC and MCID

The MDC expresses the minimal magnitude of change above which the observed change is likely to be real and not just measurement error. To estimate MDC, the standard error of measurement (SEM), which represents the amount of error associated with the assessment of an individual [38], was estimated first using the formula: ${SD}_{T1}\times \sqrt{1-R}$, where *SD*_*T*1_ is the SD of the sample at baseline and *R* is the reliability coefficient. Cronbach’s α was used as a reliability measure [39]. From the SEM, the MDC was derived as follows [38]: $MDC= SEM\times z- score\times \sqrt{2}$. A 95% confidence level (MDC_95%_) was established, corresponding to a z-value of 1.96: if a patient has a change score at or above the MDC_95%_ threshold, it is possible to state with 95% confidence that this change is reliable and not the result of a measurement error.

To assess the usefulness of anchor questions in establishing the MCID, we have evaluated their validity through the association between anchor question responses and the change scores in MLHFQ domains, by calculating partial correlation coefficients, controlling for baseline score. We hypothesized that these correlations should be higher than 0.50 [40].

We used two different statistical methods to calculate values for MCID. MCID reflects the smallest changes after a clinical intervention or natural progression that are meaningful for the patient. First, the MCID was estimated for MLHFQ considering the mean change score for patients whose response to the corresponding anchor question was “somewhat better” [41]. For the total score, this group corresponded to those patients whose response to one anchor question was “somewhat better” and to the other was “about the same”, and those whose response to both questions was “somewhat better”. In addition, to calculate cut-off values for MCID, we used the receiver operating characteristic (ROC) curve approach, considering the dichotomized anchor question responses (improved vs the same or worsened) as the dependent variable, and the change score for each dimension as an independent variable. For each dimension, the cut-off that maximized the sum of sensitivity and specificity was considered the optimal cut-off.

Further, we estimated the MCID and MDC proportions, which are the proportions of the sample with change scores exceeding the MCID and MDC_95%_, respectively. Finally, the MCID was divided by the MDC_95%_ to determine whether the MCID exceeded the MDC_95%_ [27]. If this ratio is greater than 1, the MCID can be discriminated distinguished from measurement error.

All effects were considered statistically significant at *P <* 0.05. All statistical analyses were performed using SAS 9.2 (SAS Inc., Cary, NC) and IBM SPSS Statistics for Windows, Version 23.0 (IBM Corp; Armonk, NY).

## Results

### Descriptive statistics

During the recruitment period, 2565 patients hospitalized for HF fulfilled the selection criteria, agreed to participate and completed the baseline questionnaires. Of these, 416 died within 6 months, and of the remaining 2149 patients, 1211 (56.36%) completed the questionnaires 6 months after discharge. Table 1 shows descriptive statistics for sociodemographic, clinical and HRQoL data at baseline of responders and non-responders at 6 months. There were no statistically significant differences between responders and non-responders in body mass index or smoking history. In contrast, non-responders were more likely to be older (78.45 vs. 75.92), to be women (52.51% vs. 46.49%), to have a left ventricular ejection fraction > 45% (63.65% vs. 57.85%), to be in NYHA class III or IV at discharge (50.29% vs. 37.89%) or and to have a high score (> 3) in the Charlson comorbidity index (23.71% vs 19.49%). The distribution of the MLHFQ subscales at baseline reach the possible range; psysical subscale from 0 to 40, emotional subscale from 0 to 25 and total scale from 0 to 105. In responders, the mean (SD) of the MLHFQ baseline scores were 26.14(9.66), 11.73 (7.18) and 55.45 (23.66) for the physical, emotional and total scale. The non-responders had the mean scores significantly higher in all MLHFQ domains.

**Table 1:** Sociodemographic, clinical, and Minnesota Living with Heart Failure Questionnaire (MLHFQ) baseline descriptive statistics

Parameter	Responders *n* = 1211	Non-responders *n* = 1354	*p* value
	*N* (%)	*N* (%)	
Age, mean (SD), years	75.92 (10.33)	78.45 (9.96)	< 0.0001
Gender, women	563 (46.49)	711 (52.51)	0.002
BMI, kg/m^2^			
BMI < 25	110 (20.60)	124 (20.36)	0.812
25 ≤ BMI < 30	203 (30.01)	223 (36.62)	
30 ≤ BMI < 35	141 (26.40)	158 (25.94)	
BMI ≥ 35	80 (14.98)	104 (17.08)	
Smoking history			
No	595 (56.67)	637 (59.64)	0.380
Ex	365 (34.76)	345 (32.30)	
Yes	90 (8.57)	86 (8.05)	
Left ventricular ejection fraction			
≤ 45%	462 (42.15)	426 (36.35)	0.005
> 45%	634 (57.85)	746 (63.65)	
NYHA classification at discharge			
I	62 (5.12)	30 (2.22)	< 0.0001
II	707 (58.38)	643 (47.49)	
III	425 (35.09)	649 (47.93)	
IV	17 (1.40)	32 (2.36)	
Charlson comorbidity index			
0	94 (7.76)	92 (6.79)	0.047
1	283 (23.37)	270 (19.94)	
2	323 (26.67)	367 (27.10)	
3	275 (22.71)	304 (22.45)	
> 3	236 (19.49)	321 (23.71)	
MLHFQ baseline scores, mean (SD)			
Physical subscale	26.14 (9.66)	28.42 (8.36)	< 0.0001
Emotional subscale	11.73 (7.18)	12.99 (7.32)	< 0.0001
Total scale	55.45 (23.66)	59.73 (21.62)	< 0.0001

Data are expressed as frequency (percentage) unless otherwise stated. Percentages exclude patients with missing data

*SD* Standard deviation, *BMI* Body mass index, *NYHA* New York Heart Association

MLHFQ physical subscale scores range from 0 to 40, emotional subscale scores from 0 to 25 and total scores from 0 to 105, with higher scores indicating worse health status

### Statistically significant change

All the MLHFQ scales showed minor floor and ceiling effects (< 15%) both at baseline and at six months after discharge. The mean changes in all MLHFQ domains followed a gradient (*P* < 0.0001) with larger gains in quality of life among patients classified as “improved”, smaller gains among those classified as “the same”, and losses among those classified as “worsened” (Table 2). Six months after discharge, the MLHFQ physical, emotional, and total scores decreased 10.41, 4.07 and 21.83 points, respectively, among patients classified as “improved”, all of these changes being statistically significant (*P* < 0.0001). Among patients classified as “worsened”, losses in quality of life were detected in all MLHFQ domains, with negative mean changes, although the changes in the total scale were not significant. Among patients classified as “the same”, changes were also significant in all MLHFQ domains (*P* < 0.0001) but were smaller than the changes in “improved” patients.

**Table 2:** Responsiveness parameters for the Minnesota Living with Heart Failure Questionnaire (MLHFQ) scales 6 months after discharge according to transitional questions (*n* = 1211)

	Physical			Emotional			Total		
	Improved^a^ (*n* = 393)	The same^b^ (*n* = 335)	Worsened^c^ (*n* = 238)	Improved^a^ (*n* = 280)	The same^b^ (*n* = 451)	Worsened^c^ (*n* = 234)	Improved^a^ (*n* = 397)	The same^b^ (*n* = 270)	Worsened^c^ (*n* = 296)
Mean (SD)									
At baseline	26.46 (9.31)	25.67 (9.59)	27.30 (9.40)	11.63 (7.11)	11.25 (7.20)	13.36 (7.16)	55.80 (22.60)	53.35 (22.99)	58.26 (24.15)
At 6 months	15.90 (11.08)	20.64 (11.55)	28.75 (9.57)	7.34 (6.34)	9.26 (7.18)	15.95 (6.69)	33.41 (23.65)	41.67 (25.11)	60.92 (23.79)
Change	10.41^bc^ (12.46)	5.03^ac^ (11.29)	−1.52^ab^ (10.18)	4.07^bc^ (8.00)	1.91^ac^ (8.00)	−2.59^ab^ (7.53)	21.83^bc^ (27.51)	11.39^ac^ (26.09)	−2.44^ab^ (25.50)
*P* value^*^	< 0.0001	< 0.0001	0.043	< 0.0001	< 0.0001	< 0.0001	< 0.0001	< 0.0001	0.165
SES (CI 95%)	1.12 (0.94–1.29)	0.52 (0.40–0.68)	0.16 (0.02–0.30)	0.57 (0.43–0.72)	0.27 (0.15–0.36)	0.36 (0.24–0.50)	0.97 (0.84–1.11)	0.50 (0.37–0.63)	0.10 (0.01–0.22)
SRM (CI 95%)	0.84 (0.71–0.97)	0.45 (0.33–0.54)	0.15 (0.02–0.27)	0.51 (0.38–0.63)	0.24 (0.15–0.34)	0.34 (0.21–0.48)	0.79 (0.65–0.90)	0.44 (0.33–0.56)	0.10 (0.02–0.20)

^*^Paired *t-*test to compare the mean scores at baseline and at 6 months after discharge

*SD* standard deviation, *SES* standardized effect size, *SRM* standardized response mean, *CI* 95% confidence interval

MLHFQ physical subscale scores range from 0 to 40, emotional subscale scores from 0 to 25, and total scores from 0 to 105, with higher scores indicating worse health status. Changes were calculated by subtracting follow-up scores from baseline scores; a positive result indicates a gain in quality of life

^abc^ Superscript letters indicated differences among the three subgroups (improved, equal, and worsened) according to Scheffe’s test for multiple comparisons at *P* < 0.05

### SES and SRM

The SES and SRM responsiveness parameters in “improved” patients were higher than 0.80 for the physical and total scale, but the scores values were 0.57 and 0.51 for the MLHFQ emotional scale. Among patients classified as “worsened”, effect sizes were below 0.40, while among patients classified as “the same”, values ranged from 0.52 to 0.24.

### MCID and MDC

MDC ranged from 7.27 for the emotional domain to 16.96 for the total score of the MLHFQ (Table 3). The MDC proportion ranged from 22.86% in the emotional domain to 41.10% in the total score. Controlling for baseline scores, the correlation between anchor question responses and change scores in MLHFQ domains was 0.464 in the physical domain, 0.414 in the emotional domain and 0.430 in the total scale.

**Table 3:** Responsiveness parameters for the Minnesota Living with Heart Failure Questionnaire (MLHFQ) scales at 6 months after discharge (*n* = 1211)

	MLHFQ		
	Physical	Emotional	Total
Cronbach’s α	0.89	0.87	0.93
SEM	3.00	2.62	6.12
MDC at the 95% confidence level	8.33	7.27	16.96
MDC proportion	38.07	22.86	41.10
MCID-based on transitional questions			
MCID (CI9 5%)	9.17 (7.79–10.55)	3.59 (2.52–4.66)	19.14 (16.04–22.24)
MCID proportion	35.25	37.49	36.72
Ratio of MCID to MDC at the 95% confidence level	1.10	0.49	1.13
MCID based on ROC analysis			
MCID (CI 95%)	5.00 (5.00–9.00)	1.75 (0.75–6.00)	8.20 (1.79–20.58)
MCID Proportion	51.16	46.76	53.41
Ratio of MCID to MDC at the 95% confidence level	0.60	0.24	0.48

*SEM* standard error of measurement, *MDC* minimal detectable change, *MCID* minimal clinically important difference, *CI* 95% confidence interval

MLHFQ physical subscale scores range from 0 to 40, emotional subscale scores from 0 to 25, and total scores from 0 to 105, with higher scores indicating worse health status

The MCID based on patients whose response to the anchor question was “somewhat better” ranged from 3.59 points in the emotional domain to 19.14 points in the total score. The MCID proportion based on anchor questions was similar for all domains, ranging from 35.25% in the physical domain to 37.49% in the emotional domain. The ratio of the MDC_95%_ and MCID based on anchor questions exceeded 1 for the physical domain and total score of the MLHFQ, but was less than 1 for emotional domain.

The MCID based on ROC analysis ranged from 1.75 points in the emotional domain to 8.20 points in the total score, and the MCID proportion ranged from 46.76% in the emotional domain to 53.41% in the total score. The ratio of MCID based on ROC analysis to MDC was less than 1 in all domains.

## Discussion

The results of this prospective observational study with a large sample of patients with HF offer new information about the responsiveness, MCID and MDC of the MLHFQ. This questionnaire was highly responsive capturing changes in HRQoL 6 months after discharge.

There was extensive evidence supporting responsiveness of the MLHFQ and its capacity to discriminate between different magnitudes of change in patients’ HRQoL. A systemic review with meta-analyses carried out by Garin et al. evaluate and compare data on the conceptual model and metric properties of several HF specific HRQoL instruments and conclude that they would primarily support the use of the MLHFQ [15].

The small ceiling and floor effects and the use of the full range of scores in a sample which covers the full range of severity, indicate that the instrument is likely to detect improvement or deterioration.

We have analyzed the validity and reliability of our anchor questions through correlations, as described in the literature [42, 43]. Partial correlations between anchor question responses and change scores were nearly 0.50 for all MLHFQ domains. This could be due to the anchor question for the total score not being a direct question asked to the patient, but rather a calculated response. Specifically, it was calculated by combining the responses to the other two anchor questions, and this could be expected to affect its validity and reliability.

In our study, the anchor question responses indicated that patients who reported improvement gained more points than patients who remained the same or worsened in all domains of the MLHFQ. In line with this, a large effect (SES > 0.90) was found in “improved” patients in the physical domain and total score, and a moderate effect (SES 0.57) in the emotional domain. Taken together, these findings suggest that all domains of the MLHFQ have a good sensitivity to change in our population. These results are similar to those of previous responsiveness studies in other languages [14, 18, 30, 44, 45], and what is more, our SES and SRM for “improved” patients and our change scores for improvement in all domains are larger than those reported in the other studies analyzed [14, 18, 44, 45]. The SES has also been found to be lower in the emotional domain than in the other domains in several MLHFQ responsiveness studies [14, 30, 45]. Nevertheless, in our case, this effect was moderate, being larger than SES values considered non-significant or small in other responsiveness studies.

On the other hand, in “worsened” patients, the effect size is small or not significant in nearly all domains. That is, the instrument is more responsive to improvement than worsening, not reflecting well changes in patients whose health deteriorates. These results are similar to the outcomes in the Spanish validation of the MLHFQ, the effect sizes for the patients showing “deterioration” only being > 0.26 for all domains [14]. In line with this, other studies carried out in US [45, 46] also found this questionnaire to lack discriminative power for detecting negative changes. For this reason, the MCID for “worsened” patients was not calculated. Nevertheless, for patients whose response to the anchor question was “somewhat better”, we found that changes were large enough to exceed the MDC at the individual level with a 95% level of confidence in the physical domain and total score of the MLHFQ. That is, in both cases, the change observed was greater than that required to be considered a true change, and hence, the MLHFQ can be considered responsive to detect true changes in HF patients at the individual level in the physical domain and total score.

Considering cut-off values determined by the ROC analysis, we found less conservative values for MCID, patients needing fewer points to detect such a difference. In addition, the ratio obtained from ROC analysis was not greater than 1, meaning that the change could not be distinguished from measurement error. We conclude that the MCID based on our anchor questions is more appropriate for detecting true changes.

Just like in our study, the findings of responsiveness and sensitivity of MLHFQ in other language versions show similar results. In general, patients with an improvement (measured by different ways), on overage, experienced large improvements in HRQoL. However patients with no change still experienced a moderate improvement, and those who worsened, on average, had little to no change in HRQoL [14, 18, 44–48]. Likewise, these studies confirm that the MCID in the MLHFQ exceeded predefined criteria and be more clinically valid for patients with HF than other instruments [48].

This study has various limitations that should be taken into account. Comparing responders and non-responders, those who did not respond were found to have poorer baseline MLHFQ. This might have skewed the result but any such bias would have been in our favor, as patients with a poorer health status at baseline tend to have more gains in HRQoL in follow-up. Hence, the effect size might have been smaller in our responders than it would have been in the non-responders. In addition, the differences identified may have been statistically significant due to the large sample size, while not seeming to be clinically relevant. ES are measures of the magnitude of the change scores, rather than the validity of the change scores. Therefore, ES should be considered inappropriate as parameters of the responsiveness. However, we have included these measures because they are frequently used and easily identifiable in the literature. On the other hand, we have used the Spanish version of the questionnaire, and therefore, the results may not be generalizable to other population or languages.

Nevertheless, the current study also has a number of strengths. Despite there being other studies of MLHFQ responsiveness, to the best of our knowledge, this is the first study that explores the responsiveness of the MLHFQ by estimating MDC and MCID in Spain. The present study provides data on responsiveness of the MLHFQ that could help to interpret changes detected by the questionnaire. In particular, the analysis included calculation of the MDC and MCID, a type of anchor-based method that is considered important for patients and clinicians and directly reflects their points of view [49].

Recently, Bilbao et al. [50] compared different factor structures of the MLHFQ proposed by several authors and found that their results supported the existence of a third factor, a social dimension, with good validity and reliability. They concluded that Munyombwe’s [34] model had the best psychometric properties among the social factor proposed. Unfortunately, we have not analyzed the responsiveness of this factor because we did not have an appropriate anchor question to measure it. Further studies are needed to explore the responsiveness of the social factor proposed by Munyombwe [34].

## Conclusions

To sum up, all of these results suggest that all domains of the MLHFQ have a good sensitivity to change in the population studied. It is very important to determine the responsiveness of all MLHFQ domains because these domains could reveal physiological or pathological changes, and detection of such changes could allow interventions to prevent further deterioration in heart function, and thereby reduce repeat hospitalization and mortality rates.

## Abbreviations

HF: Heart failure
HRQoL: Health related quality of life
MCID: Minimal clinically important difference
MDC: Minimal detectable change
MLHFQ: Minnesota Living with Heart Failure Questionnaire
NYHA: New York Heart Association
ROC: Receiver operating characteristics
SD: Standard deviation
SEM: Standard error of measurement
SES: Standardized effect size (SES)
SRM: Standardized response mean
